# Investigation of MicroRNA-134 as a Target against Seizures and SUDEP in a Mouse Model of Dravet Syndrome

**DOI:** 10.1523/ENEURO.0112-22.2022

**Published:** 2022-09-23

**Authors:** Rogério R. Gerbatin, Joana Augusto, Gareth Morris, Aoife Campbell, Jesper Worm, Elena Langa, Cristina R. Reschke, David C. Henshall

**Affiliations:** 1 Department of Physiology and Medical Physics, RCSI University of Medicine and Health Sciences, Dublin, D02 YN77, Ireland; 2 FutureNeuro SFI Research Centre, RCSI University of Medicine and Health Sciences, Dublin, D02 YN77, Ireland; 3 School of Pharmacy and Biomedical Sciences, RCSI University of Medicine and Health Sciences, Dublin, D02 YN77, Ireland; 4 Department of Physiology, Faculty of Medicine, Trinity College Dublin, Dublin, D02 PN40, Ireland; 5 Department of Neuroscience, Physiology and Pharmacology, University College London, London, WC1E 6BT, United Kingdom; 6 Roche Innovation Center Copenhagen, Copenhagen, CH-4070, Denmark

**Keywords:** Dravet syndrome, miR-134, oligonucleotides, seizure, SUDEP

## Abstract

Dravet syndrome (DS) is a catastrophic form of pediatric epilepsy mainly caused by noninherited mutations in the *SCN1A* gene. DS patients suffer severe and life-threatening focal and generalized seizures which are often refractory to available anti-seizure medication. Antisense oligonucleotides (ASOs) based approaches may offer treatment opportunities in DS. MicroRNAs are short noncoding RNAs that play a key role in brain structure and function by post-transcriptionally regulating gene expression, including ion channels. Inhibiting miRNA-134 (miR-134) using an antimiR ASO (Ant-134) has been shown to reduce evoked seizures in juvenile and adult mice and reduce epilepsy development in models of focal epilepsy. The present study investigated the levels of miR-134 and whether Ant-134 could protect against hyperthermia-induced seizures, spontaneous seizures and mortality (SUDEP) in F1.*Scn1a(*+/−*)^tm1kea^* mice. At P17, animals were intracerebroventricular injected with 0.1–1 nmol of Ant-134 and subject to a hyperthermia challenge at postnatal day (P)18. A second cohort of P21 F1.*Scn1a(*+/−*)^tm1kea^* mice received Ant-134 and were followed by video and EEG monitoring until P28 to track the incidence of spontaneous seizures and SUDEP. Hippocampal and cortical levels of miR-134 were similar between wild-type (WT) and F1.*Scn1a(*+/−*)^tm1kea^* mice. Moreover, Ant-134 had no effect on hyperthermia-induced seizures, spontaneous seizures and SUDEP incidence were unchanged in Ant-134-treated DS mice. These findings suggest that targeting miR-134 does not have therapeutic applications in DS.

## Visual Abstract

**Figure:**
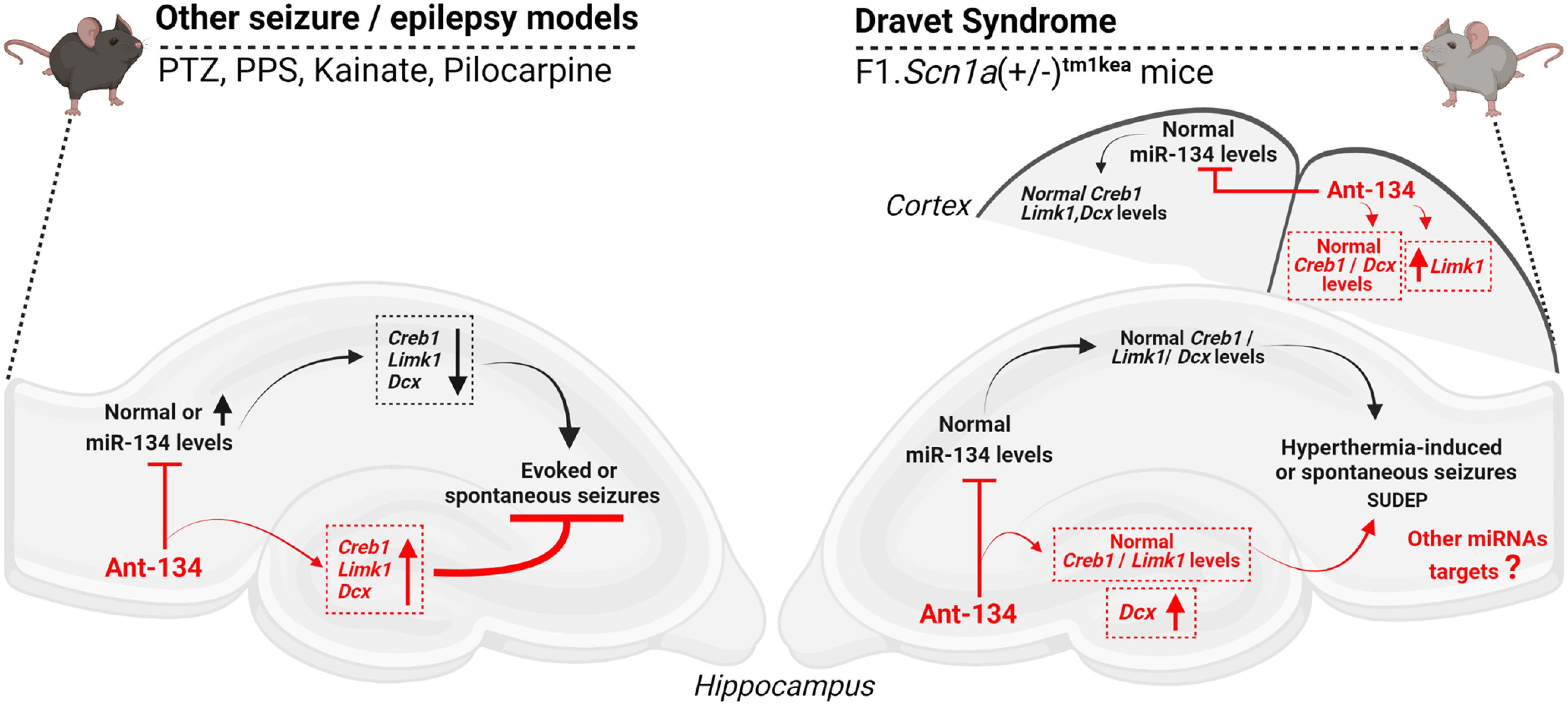

## Significance Statement

Several preclinical models of epilepsy have implicated miRNA-134 (miR-134) as a therapeutic target for seizure control and anti-epileptogenesis. The present study here explored whether targeting miR-134 has effects on seizures and mortality in a mouse model of Dravet syndrome (DS). The results indicate that suppression of miR-134 using an antimiR (Ant-134) did not protect against hyperthermia-induced seizures, spontaneous seizures or SUDEP in F1.*Scn1a(*+/−*)^tm1kea^* mice. The findings suggest that miR-134 is not a therapeutic target in DS.

## Introduction

Epilepsy is a common, chronic brain disease characterized by an enduring predisposition to generate epileptic seizures (Fisher et al., 2014). Most monogenic causes of epilepsy arise from inherited or *de novo* mutations in genes that encode protein components of ion channels or neurotransmitter systems. This includes Dravet syndrome (DS), an intractable form of childhood epilepsy with an incidence of 1:15,700 births (Wu et al., 2015). Most DS patients carry *de novo* mutations in one allele of the *SCN1A* gene leading to haploinsufficiency of the type 1 voltage-gated sodium channel α subunit (Nav1.1). Since Nav1.1 is enriched in fast-spiking parvalbumin (PV) interneurons, loss-of-function mutations in *SCN1A* result in reduced Na^+^ influx leading to reduced firing of inhibitory neurons and brain hyperexcitability (Yu et al., 2006; Jiang et al., 2018).

The earliest DS symptoms in both humans and mouse models are primarily characterized by sensitivity to hyperthermia-induced seizures (Oakley et al., 2009; Gataullina and Dulac, 2017). Severe spontaneous recurrent seizures (SRS) emerge soon after and there is a high incidence of sudden unexpected death in epilepsy (SUDEP) (Shmuely et al., 2016). Seizures in DS patients are largely refractory to current therapies. Although seizure frequency declines with age, individuals with DS often experience long-lasting cognitive and motor impairments (Genton et al., 2011; Shmuely et al., 2016). Therefore, there is an urgent need to find effective treatments for this catastrophic childhood epilepsy.

Precision therapies designed to restore SCN1A expression recently entered clinical trials (NCT04740476, NCT04442295). However, most recently approved therapies for DS remain nonprecision therapies involving broad targets to regulate brain excitability including cannabidiol, stiripentol and fenfluramine. In addition to these small molecules, a recent study using antisense oligonucleotides (ASOs) to knock-down the TAU protein in neurons reduced epilepsy, SUDEP, and autism-like behavior in a mouse model of DS (Shao et al., 2022). These studies highlight that SRS, SUDEP and behavior impairments in DS can be controlled by distinct mechanisms associated with brain excitability but not necessarily linked directly to the genetic mutation. The effectiveness of these therapies at different life-stages of DS remains unclear, however, necessitating the pursuit of additional strategies.

MicroRNAs (miRNAs) are small noncoding RNAs which have emerged as potential treatment targets in epilepsy (Brennan and Henshall, 2020; Chakraborty et al., 2021; Morris et al., 2021). Several miRNAs play crucial roles in brain development by tightly regulating post-transcriptional expression of genes implicated in brain excitability and neuronal network function (Follert et al., 2014). Among these, miRNA-134 (miR-134) is a leading candidate, a brain-enriched neuronal miRNA which has been reported to be upregulated in preclinical rodent models of seizures and epilepsy and in resected brain tissue from children and adults with drug-resistant temporal lobe epilepsy (TLE) (Jimenez-Mateos et al., 2012; Peng et al., 2013; Reschke et al., 2017).

The broad effect in multiple rodent models of seizures and epilepsy using locked nucleic acid oligonucleotide ASOs called antimiRs (Ant-134) have been linked to de-repression of structural and transcriptional proteins including Lim-domain-containing protein kinase1 (*Limk*1), Doublecortin (*Dcx*), and cAMP response element binding protein (*Creb1*), which can directly alter synaptic function and brain excitability (Schratt et al., 2006; J Gao et al., 2010; Gaughwin et al., 2011; Morris et al., 2019). Thus, Ant-134 promotes a potent seizure reduction in adult models of evoked seizures and epilepsy (Jimenez-Mateos et al., 2012; Reschke et al., 2021). Targeting miR-134 also reduced kainic acid-induced seizures, at least within a narrow dose-range, in juvenile (P21) mice (Campbell et al., 2021). Interestingly, in a mouse model of Angelman syndrome carrying a loss-of-function mutation in the maternally-inherited copy of the Ube3a gene, Ant-134 treatment also reduced effectively the susceptibility to audiogenically-evoked seizures (Campbell et al., 2022). Whether targeting miR-134 has effects in other models of genetic epilepsy is unknown, although ion channel-specific epilepsies can be treated by miRNA-targeting antimiRs in mice (Gross et al., 2016; Tiwari et al., 2019).

Here, we investigate whether miR-134 suppression would protect against hyperthermia-induced seizures, SRS and SUDEP in F1.*Scn1a*(+/−)^tm1kea^ mice by directly regulating proteins related to synaptic function and brain excitability commonly associated with the prolonged and sustained effects of Ant-134 on epilepsy. The results show that ant-134 treatment is not effective in suppressing seizures in F1.*Scn1a*(+/−)^tm1kea^ mice, suggesting that miR-134 is not able to modulate the epileptogenic process in DS and does not represent a therapeutic target for the disease.

## Materials and Methods

### Mice and ethics statement

*Scn1a^tm1Kea^* mice which have a deletion of the first coding exon were generated by homologous recombination in TL1 ES cells (129S6/SvEvTac) as previously described (Miller et al., 2014). Male *Scn1a(*+/−*)^tm1Kea^* mice on the 129S6/SvEvTac background were crossed with inbred female mice C57BL/6JOlaHsd resulting in [129XB6]F1.*Scn1a(*+/−*)* mice offspring, referred to herein as F1.*Scn1a(*+/−*)*^tm1Kea^. Both male and female F1.*Scn1a(*+/−*)*^tm1Kea^ or wild-type (WT) littermates F1.*Scn1a(+/+)*^tm1Kea^ used in experiments were genotyped before postnatal day (P)7. All animal experiments were performed in accordance with the European Communities Council Directive (86/609/EEC) and approved by the Research Ethics Committee of the Royal College of Surgeons in Ireland (REC 1302bbb) under license from the Department of Health (AE19127/P064), Dublin Ireland. Animals were maintained in a light (8 A.M. to 8 P.M.)/dark cycle (8 P.M. to 8 A.M.) with food and water *ad libitum*.

### Intracerebroventricular injections of antimiRs

At P17, mice were weaned and injected intraperitoneally with buprenorphine (0.3 mg/ml) and placed in an adapted stereotaxic frame under anesthesia (isoflurane/oxygen 5% for induction and 3% for maintenance). Body temperature was maintained by a feedback-controlled heat blanket. After topical application of EMLA cream 5%, a midline scalp incision was performed and a craniotomy was drilled to allow direct ICV injections [coordinates from bregma: anterior–posterior (AP) = +0.3 mm, lateral (L) = +0.9 mm, ventral (V) = −1.35 mm relative to the dura mater; see Extended Data Fig. 1-1 for representative images of intracerebroventricular injections in P21 mice]. Mice were randomly assigned into 5 different groups: (+/+)scr 1 nmol, (+/−)scr 1 nmol, (+/−)Ant-134 0.1 nmol, (+/−)Ant-134 0.5 nmol, and (+/−)Ant-134 1 nmol to receive three different doses of mmu‐miR‐134‐5p miRCURY LNA inhibitor (Ant-134; Exiqon; 0.1, 0.5 or 1 nmol in 2 μl PBS) or a nontargeting scrambled control (scr; Exiqon, 1 nmol in 2 μl PBS) using a 2-μl Hamilton syringe at a rate of 1 μl/min. After surgery, animals were immediately placed in an incubator at 33°C and monitored for 30 min before returning to the home cage.

### Ant-134 testing on hyperthermia-induced seizures during the febrile stage of DS

At P18, animals were subjected to a hyperthermia-induced seizure threshold assay as previously described (Gerbatin et al., 2022). First, the mouse was gently hand-restrained in a supine position with tail lifted. Then, a temperature probe (RET-4, physitemp) covered with Vaseline was inserted into the rectum and taped on the tail, to keep it in place throughout the procedure. Then, animals were placed into a Plexiglas box with an infrared heat lamp (HL-1, physitemp) positioned above and the rectal probe attached to a TCAT-2DF thermocontroller (physitemp). Mice were held at 37.5°C for 5 min to become accustomed to the chamber. Then core body temperature was gradually elevated by 0.5°C every 2 min until a seizure occurred or until reaching 42.5°C. If reaching that temperature, animals were held for 3 min before turning off the heat lamp. After that, mice remained 5 min in the chamber for observation of any late occurring seizures before they were removed, cooled down and considered seizure free. If the mouse had a seizure during the hyperthermia challenge, the heating process was stopped immediately to cool down the mouse to 37°C on a cold metal surface. Seizure severity was classified according to the Racine scale scoring system with few modifications (Racine, 1972; Van Erum et al., 2019). No behavior changes (0), mouth and facial movements (1), head nodding (2), unilateral forelimb clonus (3), bilateral forelimb clonus with rearing (4), rearing and falling (loss of posture; 5), wild running or jumping (6), and tonic hindlimb extension possibly leading to death (7).

### Measurement of miR-134 and antimiR knock-down

To evaluate miR-134 levels in the febrile stage, animals received an intraperitoneal overdose of pentobarbital 30 min after the hyperthermia challenge to be transcardially perfused with ice-cold PBS to remove the brain and microdissect the cortex and hippocampus for molecular analyses. During the worsening stage of DS, a subgroup of mice intracerebroventricularly injected at P21 with Ant-134 0.1 nmol, were also euthanized 24 h later (at P22) to evaluate the silencing of miR-134 in cortex and hippocampus. RNA was extracted from the ipsilateral hippocampus and cortex using 750 μl of TRIzol (Sigma-Aldrich), to homogenize the samples followed by a centrifugation at 12,000 × *g* for 10 min at 4°C. Phase separation was performed by adding 200 μl of chloroform (Sigma-Aldrich), to each sample and vigorously mixing for 15 s before incubating at room temperature for 5 min. Samples were centrifuged at 15,600 × *g* for 15 min at 4°C. The upper phase was removed and 450 μl of isopropanol (Sigma-Aldrich), was added and samples were stored at −20°C overnight. Samples were centrifuged at 15,600 × *g* for 15 min at 4°C and 750 μl of 75% cold ethanol (Sigma-Aldrich), was used to wash the pellet. Samples were centrifuged at 13,300 × *g* for 5 min and the ethanol was removed. Finally, pellets were left to dry for 1 h and resuspended in 25 μl of RNase free H_2_0. The quantity and quality of RNA were measured using a Nanodrop Spectrophotometer (Thermo Fisher Scientific). Samples with a 260/280 ratio between 1.8 and 2.2 were considered acceptable; 250 ng of total RNA was used to produce cDNA by reverse transcription using Multiscribe reverse transcriptase enzyme (Invitrogen).

Generated cDNA was diluted with nuclease-free dH_2_O in a ratio of 1:10. 1 μl of diluted cDNA was then mixed with a master mix containing 5 μl 2× TaqMan Fast Universal PCR Master Mix, 0.5 μl mmu-miR-134 (Applied Biosystems miRNA assay ID 001186, primer UGUGACUGGUUGACCAGAGGGG) and 3.5 μl of dH_2_O. Samples were added in triplicates in a 96-well plate. The plate was then covered with an optical adhesive film (MicroAmp, Applied Biosystems) and briefly centrifuged at 1000 × *g* for 1 min and placed in the QuantStudio 12K Flex PCR system. Comparative CT values were measured. MiRNA levels were normalized using RNU19 (Applied Biosystems miRNA assay ID 001003) expression and relative fold change in miRNA levels were calculated using the comparative cycle threshold method (2-ΔΔCT).

### Analysis of mRNA expression

qPCR was performed using the QuantiTech SYBR Green kit (QIAGEN) and the LightCycler 1.5 (Roche Diagnostics). Each reaction tube contained 2 μl cDNA sample, 10 μl SYBR Green QuantiTech Reagent (QIAGEN), 1.25 m primer pair (Sigma-Aldrich), and RNase free water (Invitrogen) to a final volume of 20 μl. Using LightCycler 1.5 software, data were analyzed and normalized to the expression of -actin. Primers used (Sigma-Aldrich) were as follows: β-actin forward 5′-AGGTGTGATGGTGGGAATGG, reverse 5′-GGTTGGCCTTAGGGTTCAGG; *Limk1* forward, 5′-TTATCGGGCGTGTGAATGCA, reverse 5′-ACCAGACAAGTGCATGGGAA; *Creb1* forward 5′-TGGGGACTGGCATTTTGTA, reverse 5′-GCAGGAGAAAGCACAGCAAA; *Dcx* forward, 5′-GGAGTGGGTTACATTTACACCAT, reverse 5′-GTCTGAGGAACAGACATAGCTT.

### Video EEG recordings of spontaneous seizures and SUDEP during the worsening stage of DS

At P21, another cohort of F1.*Scn1a(*+/−*)^tm1kea^* mice underwent surgery as above to be randomly intracerebroventricularly injected with (Ant-134 0.1nmol in 2 μl PBS) or a nontargeting scrambled control (scr; Exiqon, 0.1 nmol in 2 μl PBS). Three screw electrodes were implanted to allow for EEG recordings and secured with dental cement and surgical glue. The screw electrodes were placed bilaterally to the midline over the cerebral cortex followed by the reference electrode positioned over the nasal sinus. After surgery, animals were immediately placed in an incubator at 33°C and monitored for 30 min. Once fully recovered, single housed mice were connected to the lead socket of a swivel commutator, which was connected to a brain monitor amplifier for EEG digital recordings. Gel diet was added in the cage and vEEG recordings were performed from 12:30 P.M. to 6:30 P.M. (6 h/d) followed by video monitoring from 6:30 P.M. to 12:30 P.M. (18 h/d) until P28. Immediately after acute vEEG recordings, single housed mice in their home cages were transferred to a room equipped with a high resolution, infrared video cameras (Hikvision). Continuous digital videos were recorded at 30 fps and stored in a Dell PC workstation. Video recordings were reviewed offline at 16× speed using VSplayer software (version 6.0.0.4) and suspected seizures were reviewed at 1× speed. Duration of seizures were defined from the beginning to end of the behavioral convulsion. The severity of spontaneous seizures was scored based on a new revised Racine Scale (Van Erum et al., 2019): normal behavior (0), generalized tonic-clonic seizure (GTCS), rearing, clonus and loss of balance/posture/falling (5), GTCS + wild running and/or jumping (6) and GTCS ending with full tonic hindlimb extension (180° relative to torso) possibly leading to cardiorespiratory arrest and death (7). Seizure severity assessment was limited to scores (0, 5, 6, and 7) to ensure consistency of analyses. When a mouse was found dead in a cage, the video was reviewed to determine whether it was preceded by a severe GTCS ending with full hindlimb extension.

### Statistical analyses

The normality of the data was analyzed using D’Agostino and Pearson’s omnibus normality test. Data were analyzed using one-way ANOVA, Kruskal–Wallis test (followed by two-stage step-up method of Benjamini, Krieger, and Yekutiel correction for multiple comparisons), unpaired two-tailed Student’s *t* test, Mann–Whitney *U* test and Kaplan–Meier method followed by Tukey’s *post hoc* test, as appropriate. Grubbs’ test with α = 0.01 was applied to detect outliers in mRNA levels of *Dcx*. The specific statistical test used for each experiment are indicated in the figure legends. Data are expressed as SDs or median with interquartile range (IQR), as appropriate. Differences between groups were considered statistically significant when *p* < 0.05. Experiments and data were analyzed blind to genotype and treatment. Further information of each statistical test performed are showed in Table 1.

## Results

### Evaluation of Ant-134 on hyperthermia-induced seizures in F1.*Scn1a(*+/−*)^tm1kea^* mice

**Figure 1.**
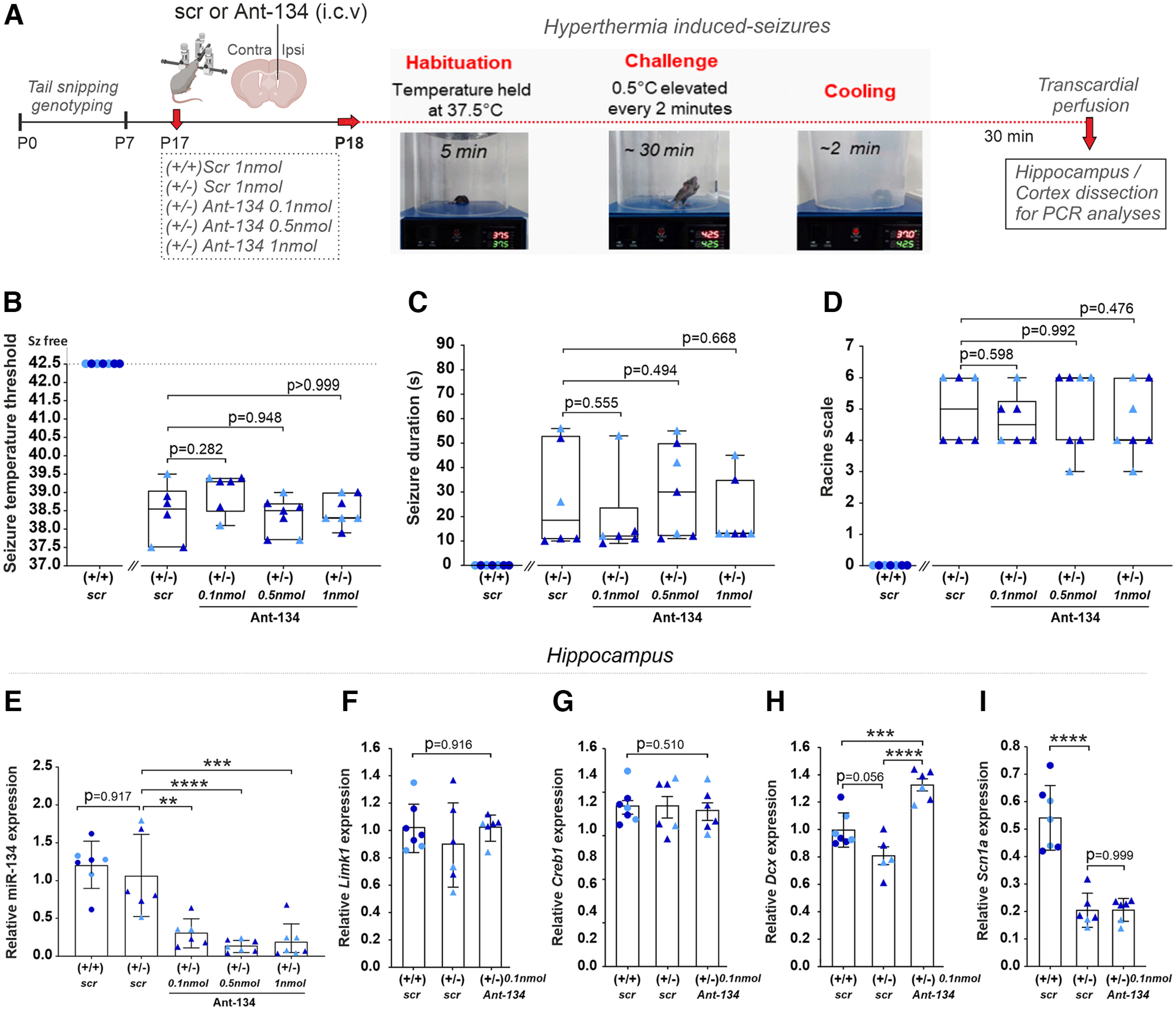
Ant-134 dose response curve on hyperthermia-induced seizures in F1.*Scn1a(*+/−*)^tm1kea^* mice. ***A***, At P17, mice were weaned and intracerebroventricularly injected with 0.1, 0.5, or 1 nmol dose of Ant-134 or scr. At P18, hyperthermia challenge was induced by placing the animals in a thermostat-controlled heating chamber. Graphs show the temperature threshold for mice to develop a seizure (***B***), median duration of seizures (***C***), and the respective seizure severity according to a modified Racine scale (***D***). ***E***, Transcript levels of miR-134 in the ipsilateral hippocampus 24 h after scr or Ant-134 injections. ***F*–*I***, Expression of validated targets of miR-134 (*Limk1*, *Creb1*, and *Dcx*) and *Scn1a* levels in scr or Ant-134-treated mice. ***B–D***, Kruskal–Wallis test, median IQR. ***E–I***, One-way ANOVA, mean (SD). **p < 0.01, ***p < 0.001, ****p < 0.0001. One outlier from Ant-134 F1.*Scn1a*(+/−)^tm1kea^-treated mice group was removed according to Grubbs’ test (α = 0.01) in *Dcx* transcript levels. Representative images of intracerebroventricular injections with methylene blue in P21 mice can be found in Extended Data Figure 1-1. Relative expression of miR-134, its validated targets (*Limk1*, *Creb1*, and *Dcx*) and *Scn1a* levels in cortical tissue can be found in Extended Data Figure 1-2.

Sensitivity to hyperthermia is a hallmark of DS onset. In the first experiment, we investigated whether the silencing of miR-134 by Ant-134 could prevent the development of hyperthermia-induced seizures in F1.*Scn1a(*+/−*)^tm1kea^* mice during the febrile stage of DS. A total of six to seven mice per group were used and data were analyzed by Kruskal–Wallis test. At P17, F1.*Scn1a(*+/−*)^tm1kea^* mice were intracerebroventricularly injected with 0.1, 0.5, or 1 nmol dose of Ant-134 followed by the hyperthermia challenge at P18 (Fig. 1*A*). As body temperature was elevated, all scr F1.*Scn1a(*+/−*)^tm1kea^* mice developed seizures at temperatures ranging from 37.5°C to 39.5°C (*p* = 0.0009; Fig. 1*B*) presenting a median duration ∼20 s (*p* = 0.003; Fig. 1*C*) and severity 5 according to a modified Racine scale score (*p* = 0.0006; Fig. 1*D*). Notably, none of three different doses of Ant-134 influenced the temperature threshold for seizure development (Fig. 1*B*). The duration and severity of seizures also remained unchanged in F1.*Scn1a(*+/−*)^tm1kea^* Ant-134-treated mice when compared with F1.*Scn1a(*+/−*)^tm1kea^* scr mice (Fig. 1*C*,*D*).

10.1523/ENEURO.0112-22.2022.f1-1 Extended Data Figure 1-1 Representative images of intracerebroventricular injections in P21 mice. Methylene blue into the ventricles of (***A***) male and (***B***) female P21 mice. Download Figure 1-1, TIF file.

**Figure 2.**
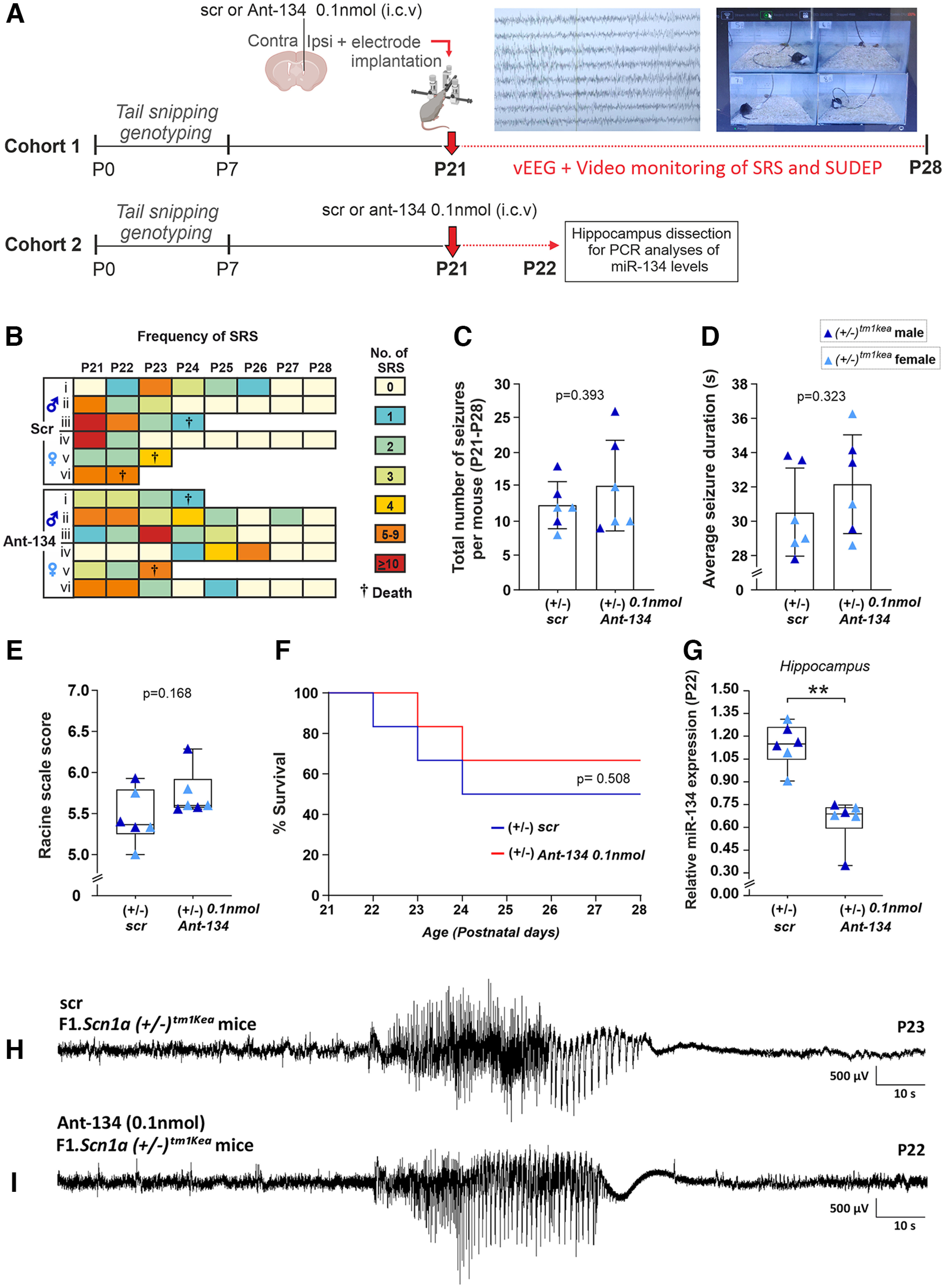
Ant-134 0.1 nmol does not prevent SRS and SUDEP occurrence in F1.*Scn1a*(+/−)^tm1kea^ mice. ***A***, Schematic showing the experimental design used to investigate the occurrence of SRS, SUDEP and miR-134 levels in F1*.Scn1a*(+/−)^tm1kea^ mice during the worsening stage of DS. ***B***, Representation of the frequency of SRS experienced by scr and Ant-134 F1.*Scn1a*(+/−)^tm1kea^-treated mice according to a color scale ranging from 0 to 10 seizures or more. ***C***, Quantitative analyses of SRS between P21 and P28. ***D***, ***E***, Seizure duration and severity in Ant-134-treated mice compared with scr. ***F***, SUDEP rates between scr and Ant-134 F1.*Scn1a*(+/−)^tm1kea^-treated mice. ***G***, Taqman results confirming Ant-134 0.1 nmol produced a knock-down in miR-134 levels when compared with scr mice. ***H***, ***I***, EEG representative trace of SRS in scr and Ant-134 F1.*Scn1a*(+/−)^tm1kea^-treated mice. (*n* = 6/group). ***C***, ***D***, Student’s *t* test, mean (SD). ***E***, ***G***, Mann–Whitney test, median IQR. ***F***, log-rank test. **p < 0.01.

10.1523/ENEURO.0112-22.2022.f1-2 Extended Data Figure 1-2 Ant-134 0.1nmol effect on miR-134, *Limk1, Creb1, Dcx* and *Scn1a* expression in the ipsilateral cortex of F1.*Scn1a*(+/-)^tm1kea^ mice. ***A***, Graph shows the miR-134 levels in the ipsilateral cortex and (***B–E***) the expression of validated targets of miR-134 *Limk1, Creb1 and Dcx* and the respective *Scn1a* levels ∼24 hrs after scr or Ant-134 0.1nmol intracerebroventricular injections. ***A–E***, One-way ANOVA, mean (SD); **p < 0.01, ****p < 0.0001. Download Figure 1-2, TIF file.

### miR-134, *Creb1, Limk1, Dcx*, and *Scn1a* expression after hyperthermia-induced seizures in F1.*Scn1a(*+/−*)^tm1kea^* mice

Next, transcript levels of miR-134 in the ipsilateral cortex and hippocampus were investigated 24 h after Ant-134 injections (Fig. 1*E*; Extended Data Fig. 1-2). All results reported here were analyzed by one-way ANOVA with a total of six to seven mice per group. No statistical difference in the levels of miR-134 in cortex or hippocampus was observed between WT controls and F1.*Scn1a(*+/−*)^tm1kea^* mice, confirming miR-134 is not differentially expressed in the DS mouse model (Fig. 1*E*; Extended Data Fig. 1-2*A*). F1.*Scn1a(*+/−*)^tm1kea^* treated mice with 0.1, 0.5, and 1 nmol dose of Ant-134 showed a miR-134 knock-down of 71.7%, 86.9%, and 82.5% respectively, when compared with scr F1.*Scn1a(*+/−*)^tm1kea^* mice (*p* = 0.001, *p* < 0.0001, and *p* = 0.0002, respectively; Fig. 1*E*). Cortical and hippocampal levels of miR-134 targets including *Limk1*, *Creb1*, and *Dcx* were also investigated (Fig. 1*F–H*; Extended Data Fig. 1-2*B–D*). No statistical difference was observed in the levels of all miR-134 targets between WT scr and F1.*Scn1a(*+/−*)^tm1kea^* scr mice in both brain regions (Fig. 1*F–H*; Extended Data Fig. 1-2*B–D*). In contrast, hippocampal levels of *Dcx* and cortical *Limk1* levels were upregulated in F1.*Scn1a(*+/−*)^tm1kea^* mice treated with ant-134 0.1 nmol when compared with scr F1.*Scn1a(*+/−*)^tm1kea^* mice (*p* < 0.0001 and *p* = 0.009, Fig. 1*H* and Extended Data Fig. 1-2*B*, respectively). These findings confirm that inhibiting miR-134 upregulates some, but not all, miR-134 targets in DS model mice. Lastly, we investigated whether Ant-134 0.1 nmol could have any effect on *Scn1a* transcript levels in DS mice. However, similar *Scn1a* levels were observed between scr F1.*Scn1a(*+/−*)^tm1kea^* and Ant-134 F1.*Scn1a(*+/−*)^tm1kea^* treated mice in both brain regions (Fig. 1*I*; Extended Data Fig. 1-2*E*).

### Ant-134 does not change the frequency of spontaneous seizures and SUDEP in F1.*Scn1a(*+/−*)^tm1kea^* mice

Some therapeutics may fail against hyperthermia-induced seizures but nevertheless work against spontaneous seizures and SUDEP (Hawkins et al., 2017). Previous work showed that Ant-134 when injected at 0.1 nmol reduced seizures evoked by systemic kainic acid in P21 mice (Campbell et al., 2021). Based on this, using a second cohort of mice, we investigated whether Ant-134 dose (0.1 nmol) injected at P21 would reduce the frequency of spontaneous seizures and SUDEP (recorded by vEEG and video monitoring) experienced by F1.*Scn1a(*+/−*)^tm1kea^* mice until P28 during the worsening stage of DS (Fig. 2*A*, *n* = 6 mice per group).

Figure 2*B*,*C* shows the frequency of spontaneous seizures and SUDEP for scr and Ant-134-treated F1.*Scn1a(*+/−*)^tm1kea^* mice. Student’s *t* test revealed no difference in the total number of spontaneous seizures over the period between scr and Ant-134-treated F1.*Scn1a(*+/−*)^tm1kea^* mice (Fig. 2*C*). Furthermore, the duration and severity of spontaneous seizures were similar between both groups (Fig. 2*D*, Student’s *t* test, *E*, Mann–Whitney test). Next, we investigated whether Ant-134 had any effect on the incidence of SUDEP in F1.*Scn1a(*+/−*)^tm1kea^* mice (Fig. 2*F*). Notably, no difference was observed in survival rates in F1.*Scn1a(*+/−*)^tm1kea^* mice treated with Ant-134 when compared with control (Fig. 2*F*, log-rank test). Lastly, another cohort of mice were injected at P21 with Ant-134 (0.1 nmol) or its vehicle to assess the silencing of miR-134 in hippocampus at P22 (Fig. 2*A*,*G*, *n* = 6 mice per group). As expected, lower levels of miR-134 were observed in the hippocampus of F1.*Scn1a(*+/−*)^tm1kea^* mice treated with Ant-134 when compared with control (Fig. 2*G*). Ant-134 0.1 nmol promoted a knock-down of ∼ 57% of miR-134 levels in F1.*Scn1a(*+/−*)^tm1kea^* mice (Fig. 2*G*, Mann–Whitney test).

## Discussion

Extensive preclinical data has shown that inhibition of miR-134 is a potential treatment for drug-resistant focal epilepsy. Recent studies also showed inhibiting miR-134 can reduce evoked seizures in immature mice and reduce seizures in a genetic model of a neurodevelopmental disorder. The present study shows that miR-134 knock-down induced by an ASO antimiR does not reduce hyperthermia-induced seizures, spontaneous seizures or SUDEP in F1.*Scn1a(*+/−*)^tm1kea^* mice. These findings indicate limitations in the application of miR-134 targeting for certain genetic forms of epilepsy.

Early DS symptoms are primarily characterized by febrile seizures emerging during the first year of life (febrile stage of DS; Gataullina and Dulac, 2017). Accordingly, P18 F1.*Scn1a(*+/−*)^tm1kea^* mice intracerebroventricularly injected with scr showed sensitivity to hyperthermia-induced seizures at temperatures ∼38.5°C. Preclinical studies have shown that intracerebroventricular injection of antagomir targeting miR-134 protects against evoked seizures in multiple rodent models of epilepsy, including models of earlier-life seizures and neurodevelopmental disorders (Reschke et al., 2017; X Gao et al., 2019; Campbell et al., 2021, 2022). In the first study here, we found that suppression of miR-134 by antimiR ASOs did not affect seizure duration, severity or temperature threshold for F1.*Scn1a(*+/−*)^tm1kea^* mice. These findings suggest that miR-134 is not involved in the susceptibility to hyperthermia-induced seizures experienced by F1.*Scn1a(*+/−*)^tm1kea^* mice.

miR-134 has been found upregulated in the hippocampus of children and adults with TLE (Jimenez-Mateos et al., 2012; Peng et al., 2013). Similarly, many preclinical models of seizures and epilepsy have shown increased levels of miR-134, which has been associated with seizure susceptibility (Peng et al., 2013; Reschke et al., 2017; X Gao et al., 2019). However, miR-134 is not upregulated in all epilepsy models, including kainic acid-induced seizures in P21 mice and elevated miR-134 was observed in the cerebellum but not hippocampus in a mouse model of the neurodevelopmental disorder Angelman syndrome (Campbell et al., 2021, 2022). Here, we showed that F1.*Scn1a(*+/−*)^tm1kea^* scr mice express normal levels of miR-134 in the cortex and hippocampus. This suggests that miR-134 does not have a key role in the pathologic molecular mechanisms underlying DS.

Key miR-134 targets in epilepsy have been identified including *Limk1*, *Creb1*, and *Dcx* that regulate dendritic spine dynamics, synaptic plasticity, and direct neuronal migration after neurogenesis, respectively (Schratt et al., 2006; J Gao et al., 2010; Gaughwin et al., 2011; Morris et al., 2019). In the intra-amygdala kainate model of epilepsy in mice, the upregulation of miR-134 expression was followed by a corresponding decrease of transcript levels of *Limk1* and *Creb1* in the hippocampus (Jimenez-Mateos et al., 2012). Ant-134 produced de-repression of both genes (Jimenez-Mateos et al., 2012). In a pediatric model of status epilepticus in P21 mice, Ant-134 treatment de-repressed cortical *Dcx* levels and this effect was associated with seizure suppression (Campbell et al., 2021). Similarly, in a genetic mouse model of Angelman Syndrome, the reduced susceptibility to audiogenic-evoked seizures in Ant-134-treated N4 Ube3a^m–/p+^ mice was associated with upregulation of *Dcx* (mRNA and protein) in the hippocampus and increased *Creb1* protein in cortex (Campbell et al., 2022). Here, we found that scr F1.*Scn1a(*+/−*)^tm1kea^* mice show similar levels of miR-134 and related targets (*Limk1*,*Creb1* and *Dcx*) in comparison to the scr F1.*Scn1a(+/+)^tm1kea^* group. In contrast, Ant-134 treatment increased *Limk1* and *Dcx* levels in cortex and hippocampus of F1.*Scn1a(*+/−*)^tm1kea^* mice respectively. Furthermore, no difference in *Scn1a* expression was observed between scr and Ant-134 F1.*Scn1a(*+/−*)^tm1kea^* mice. These results suggest that the epilepsy etiology is important for whether or not Ant-134 can exert anticonvulsant effects and that upregulation of miR-134 targets (commonly associated with a potent and sustained effect on epilepsy) are not relevant for the seizure susceptibility experienced by F1.*Scn1a(*+/−*)^tm1kea^* mice.

Between P21 and P28, DS model mice display frequent and severe spontaneous seizures which are associated with SUDEP (Kalume et al., 2013). When delivered shortly after an epilepsy-inciting episode of status epilepticus, Ant-134 produces potent and lasting suppression of spontaneous seizures in different rodent models of epilepsy (Jimenez-Mateos et al., 2012; Reschke et al., 2017, 2021). Here, we found that Ant-134 treatment was not effective in suppressing spontaneous seizures and SUDEP during the worsening stage of DS in F1.*Scn1a(*+/−*)^tm1kea^* mice. Therefore, Ant-134 had no protective effects in either stage of the model (febrile or worsening stage). This was unlikely to be because of insufficient Ant-134 since its knock-down of miR-134 was confirmed using tissues from injected mice and the dose used was the same as those previously effective in other models (Jimenez-Mateos et al., 2012; Reschke et al., 2017; Campbell et al., 2021). Taken together, these results indicate that the mechanisms of ictogenesis in DS are not sensitive to miR-134 inhibition, reinforcing that DS has, if any, alternative miRNAs driving the pathophysiology of this disease.

Therefore, Ant-134 results on DS were negative and it was not because of incorrect study design or dose. Thus, these findings have helped to resolve the issue of whether this miRNA targeting approach is broadly effective in epilepsy or as appears to be the case, etiology, or syndrome-specific. While insufficient inhibitory drive is thought to be a common mechanism underlying epilepsies, differences in how this arises be it because of single gene dysfunction or broader network damage may underlie the results. That is, this specific miRNA targeting approach may better suit the complex pathophysiology of TLE rather than the more specific or singular cause in DS. One way to address this in the future would be to test Ant-134 in another genetic model. In addition, it would be timely to profile some of the genetic epilepsies for aberrant miRNA expression and target only those specific to that model or those miRNAs which directly suppress the haploinsufficient transcript.

**Table 1:** Statistical table showing the relevant information related to each statistical test performed

	Experiment	Data structure	Type of test	Power
Fig. 1*B–D*	Temperature threshold, Seizure duration and Racine scale, respectively	Non-normal distribution	Kruskal–Wallis test	95%
Fig. 1*E–I* and Extended Data Fig. 1-2	miR-134, *Limk1*, *Creb*1, *Dcx*, and *scn1a* relative expression, respectively	Normal distribution	One-way ANOVA	95%
Fig. 2*C,D*	Number of seizure and seizure duration	Normal distribution	Student’s *t* test	95%
Fig. 2*E,G*	Racine scale and miR-134	Non-normal distribution	Mann–Whitney test	95%
Fig. 2*F*	% Survival	Non-normal distribution	log-rank test	95%

## Synthesis

Reviewing Editor: Viji Santhakumar, University of California Riverside Decisions are customarily a result of the Reviewing Editor and the peer reviewers coming together and discussing their recommendations until a consensus is reached. When revisions are invited, a fact-based synthesis statement explaining their decision and outlining what is needed to prepare a revision will be listed below. The following reviewer(s) agreed to reveal their identity: Justin Botterill, Suchitra Joshi.

Synthesis: The manuscript examines the potential efficacy of antagonizing microRNA 134 (miR-134) in limiting seizures in a genetic model of epilepsy (Dravet Syndrome). In contrast to the therapeutic and anti-seizure effects of miRNA-134 antisense oligonucleotides (Ant-134) reported in models of evoked seizures (e.g., primarily with convulsants), the Authors report that Ant-134 has limited therapeutic effect in a mouse model of Dravet Syndrome. While this is a negative finding appropriate controls are in place and the data presents an important contribution to the field because it suggests that models of acquired and genetic epilepsies can differ in their therapeutic targets. Such data are within the scope of eNeuro. The reviewers uniformly agree that the quality of the writing and figures is generally good. Overall, this is a carefully designed study. The major concern is that the rationale for the study is not well justified. There is reason for targeting miR-134 in Dravet Syndrome, a genetic epilepsy due to sodium channel mutation, with little information on whether miR-134 is impacted is not adequately justified in the introduction. Although the authors reference work suggesting that miRNA antagomirs can treat channelopathies, it is unclear why they thought ant134 would work in Dravet syndrome or how it may influence generic excitability phenotypes. There are additional concerns with methodology and presentation that need to be addressed.

Specific Comments

1. The introductions needs to be substantially revised to justify the rationale for the study. The introduction, as currently formulated, presents an impression that the authors either assume or hypothesize that miR134 would be upregulated in DS based on other preclinical models of epilepsy. Without a valid association between Scn1a mutations and changes in miR134 or related pathways, the rationale for the study is weak. The authors attempt to use the efficacy of miR134 antagomirs in protecting against seizures in experimental animals with temporal lobe epilepsy (TLE) as a justification for testing the same in Scn1a mutations. However, this misses the fact that several alterations that underlie epileptogenesis in acquired TLE can be targeted by miR134 antagonism. Whether this is the case in Scn1a mutations is not indicated. miR134 antagonism could be a useful therapeutic strategy in channelopathies if miRNAs regulate the expression of the channels implicated in these defects. Yet, whether miR134 regulates the Scn1a transcript levels is unclear and not examined.

2. The manuscript would benefit from additional details regarding the stereotaxic injection coordinates with regard to the depth used for injections of Scr or ant-134. For example, is the depth of 1.35mm relative to skull surface? While there may be a need to adjust stereotaxic coordinates in young mice, 1.35mm ventral depth seems quite superficial (even when considering these experiments were done in P17-P21 mice). Based on the brain atlas, the tip of the ventricles is around 2.1mm depth in adult mice using anterior-posterior coordinates provided in the Methods. Please provide representative histology (e.g., Nissl stain) showing the injection depth is sufficient for ICV injections at P18 or P22?

3. Figure 2: Seizure behaviors scored as 5, 6, and 7 are described in the methods. Please elaborate on what behaviors were scored as 8, 9, and 10 in figure 2B.

4. Figure 1. It is unclear whether a 30 minutes post-seizure time point is sufficient to detect whether heat-evoked seizures upregulate miR-134 in (+/-)Scr mice? For example, many of the papers the Authors cite evaluated miR-134 at significantly later post-seizure timepoints than the present study (e.g., 4, 24,or 72 hrs in Jimenez-Mateos et al., 2012, Gao et al., 2019, Campbell et al., 2021). In other words, the data in Figure 1E supports that Ant-134 reduces miR-134 transcript levels 24hrs after injections, but this singular time-point does not indicate whether there is a delayed upregulation of miR-134 in Scr(+/-) mice following heat-evoked seizures. It appears that this test may have been conducted as a positive control for drug action. The authors should address or discuss the effect of the mutation on miR-134 and the timeframe.

5. Figures 1B-1D. Please justify the need to include the (+/+)Scr mice in the statistical analyses (instead of serving as a reference in the figure) since these mice don’t have heat-induced seizures and thus have 0 values on measures of seizure duration (Fig. 1C) and the Racine scale (Fig. 1D). The (+/+) Scr group are also likely to be the main reason these datasets are not normally distributed and require non-parametric statistical analyses. Does limiting analysis to +/- mice alter the statistical outcome?

6. Figure 2. The Authors record 6hrs of vEEG and 18hrs of video recordings each day from P21-P28 to quantify the frequency and severity of spontaneous seizures. While the Authors used a conservative criteria to score only severe behavioural seizures, the manuscript does not provide any representative EEG traces or data analysis that could be used to help support the conclusions drawn by the Authors (e.g., EEG power). Authors need to include EEG traces in their revised manuscript.

7. Figure 2G. The authors need to clarify the purpose/difference between Figure 2G and results shown in Figure 1E? The results in Figure 2G include two of the same groups: (+/-)Scr and (+/-)ant-134 0.1nmol which show the same result as Figure 1E (i.e., downregulation of miRNA-134 24hrs after injection of ant-134 without any notable corresponding anti-seizure effects). While the age (P18 vs P22) and seizure-type (heat-evoked, spontaneous) differ between these two figure panels, these differences are neither emphasized nor discussed.

8. The analyses for miR-134 and related targets were done exclusively in hippocampal tissue. Are similar results observed in cortical tissues? If the Authors are unable to do additional experiments, this limitation should be addressed in the discussion.

9. Line 57: DS prevalence is indicated a 1 in 17,700 births, but the paper cited (Wu et al., 2015) indicates prevalence is 1 in 15,700 births. Please double check for accuracy.

10. Line 194-195 has a repetition error= “Primers used (Sigma-Aldrich) were as follows: Primers used (Sigma-Aldrich) were as follows:”

11. Please indicate what Limk1 and Dcx are for the benefit of broader readership. Also discuss any significance of differential effect on miR inhiation on these.
