# Supplementary figures and images for: Investigation of MicroRNA-134 as a Target against Seizures and SUDEP in a Mouse Model of Dravet Syndrome
Source: eNeuro. 2022 Sep 23;9(5):ENEURO.0112-22.2022. doi: 10.1523/ENEURO.0112-22.2022 (PMC9522462; doi:10.1523/ENEURO.0112-22.2022)

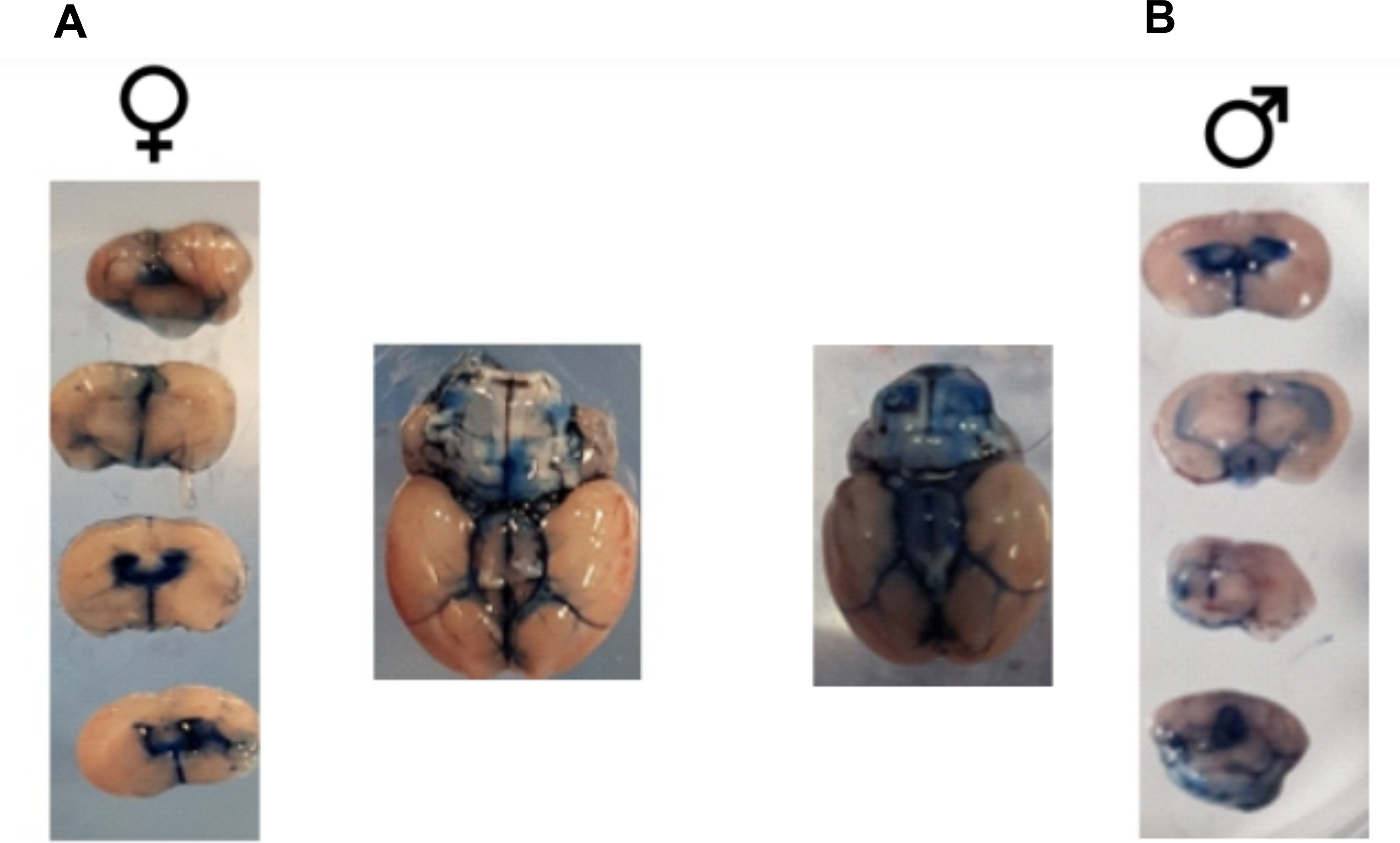

Supplement: Extended Data Figure 1-1 — Representative images of intracerebroventricular injections in P21 mice. Methylene blue into the ventricles of (A) male and (B) female P21 mice. Download Figure 1-1, TIF file. [file enu-eN-NRS-0112-22-s03.tif]

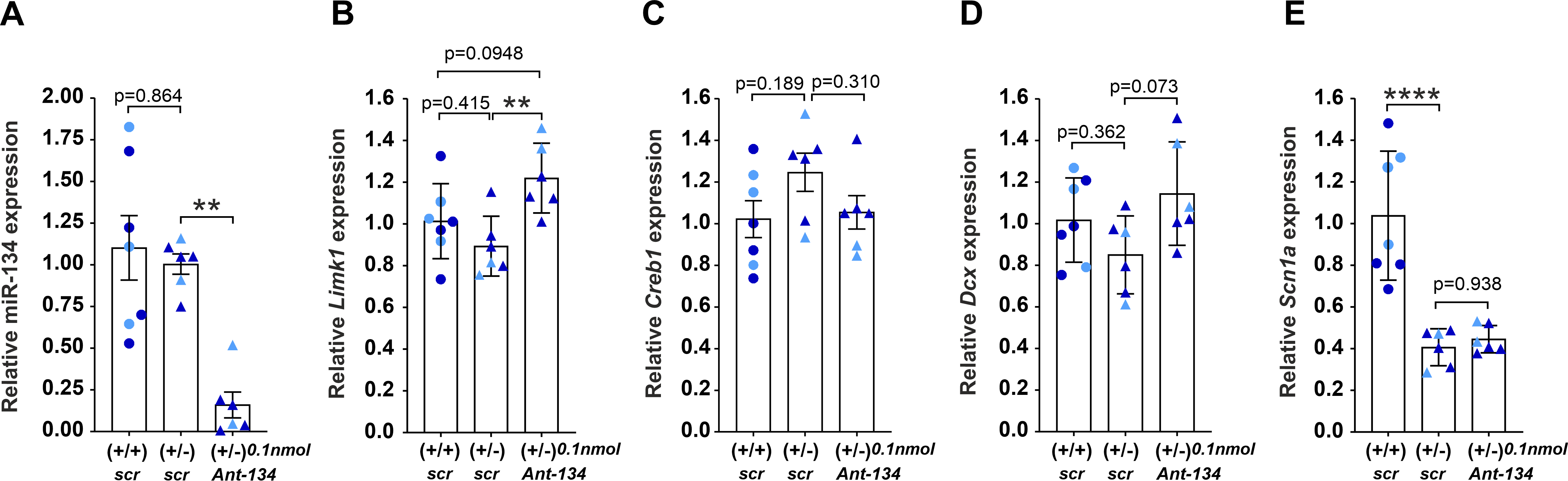

Supplement: Extended Data Figure 1-2 — Ant-134 0.1nmol effect on miR-134, Limk1, Creb1, Dcx and Scn1a expression in the ipsilateral cortex of F1.Scn1a(+/-)tm1kea mice. A, Graph shows the miR-134 levels in the ipsilateral cortex and (B–E) the expression of validated targets of miR-134 Limk1, Creb1 and Dcx and the respective Scn1a levels ∼24 hrs after scr or Ant-134 0.1nmol intracerebroventricular injections. A–E, One-way ANOVA, mean (SD); **p < 0.01, ****p < 0.0001. Download Figure 1-2, TIF file. [file enu-eN-NRS-0112-22-s04.tif]
